# Pediatric Wilson disease with early psychiatric manifestations: a case report

**DOI:** 10.1186/s13256-026-06220-7

**Published:** 2026-06-24

**Authors:** Firas Shammas, Mohammad Hamama, Sawsan Ismail, Ali Naef Alhailony, Raneem Al Dali, Solaiman Naser Solaiman

**Affiliations:** 1 Faculty of Medicine, Al Andalus University, Al Qadmūs, Syrian Arab Republic; 2 Faculty of Dentistry, Al Andalus University, Al Qadmūs, Syrian Arab Republic; 3 Department of Pathology, Faculty of Medicine, Al Andalus University, Al Qadmūs, Syrian Arab Republic; 4 Department of Neurology, National Hospital of Masyaf, Masyaf, Syrian Arab Republic; 5 Otorhinolaryngology Department, National Hospital of Masyaf, Masyaf, Syrian Arab Republic; 6 Department of Pediatrics, National Hospital of Masyaf, Masyaf, Syrian Arab Republic; 7 Syrian Medical Students’ Association, Al Sleimania, Aleppo, Syrian Arab Republic

**Keywords:** Wilson disease, Hepatic, Neuropsychiatric symptoms, Pediatric, Case report

## Abstract

**Background:**

Wilson’s disease is an autosomal recessive disorder of copper metabolism that primarily affects the liver and brain, with onset typically in early adulthood. Pediatric presentations are rare, and the atypical presentation of psychiatric symptoms can delay the diagnosis process in low-resource settings. Epidemiological data on the disease, particularly in Syria, remain scarce. Early diagnosis is necessary to prevent irreversible hepatic and neurologic damage.

**Case presentation:**

An eight-year-old Syrian child presented with psychiatric and neurologic symptoms, including increased aggressiveness, learning difficulties, dysarthria, drooling while eating and drinking, and difficulty speaking, followed by weight loss and jaundice. A dental examination revealed no temporomandibular joint deformities, prompting neurological consultation. The neurological examination revealed difficulty walking with limb tremors and speech impairment. Laboratory findings showed slightly elevated transaminases and total iron-binding capacity with low serum iron, indicating hepatic dysfunction. Serum ceruloplasmin was markedly reduced (3.1 mg/dl), and 24-h urinary copper excretion was elevated. MRI of the brain revealed increased T2-weighted signal intensity in the caudate and lentiform nuclei. An ophthalmologic exam found Kayser–Fleischer rings at the corneal limbus. Esophagogastroduodenoscopy showed distal esophageal varices, and an abdominal ultrasound showed hepatomegaly and splenomegaly. The patient was treated with copper chelator penicillamine, dietary copper restriction, and physical therapy. Follow-up showed marked clinical improvement with recovery of motor function, improved speech, and resolution of jaundice.

**Conclusions:**

This instance underscores the necessity of evaluating Wilson’s illness in pediatric patients exhibiting early behavioral and neurological signs, particularly emphasizing that prominent psychiatric manifestations may precede hepatic signs even in young children. Early administration of chelation therapy can lead to excellent outcomes even in resource-limited settings. Atypical presentations require increased awareness that may facilitate earlier detection and the prevention of irreversible organ damage.

## Background

Wilson’s disease (WD) is an autosomal recessive disease caused by a mutation in the ATP7B gene, which is responsible for impaired biliary copper excretion and the accumulation of copper in the liver and brain. The prevalence of Wilson’s disease is reported to be approximately 1 in 30,000 live births, with a typical delay in diagnosis of 1–2 years [1]. The recognition of Kayser-Fleischer rings with decreased serum ceruloplasmin and the presentation of hepatic symptoms all lead to the diagnosis of classic Wilson disease [2]. The second most common neurologic symptoms include ataxia, dysarthria, and hypersalivation. Psychiatric manifestations affect only approximately 10–20% of patients affected by WD in all age groups; pediatric data may differ [3].

## Case presentation

An 8-year-old Syrian child presented with psychiatric and neurologic symptoms, including increased aggressiveness, learning difficulties, dysarthria, drooling while eating and drinking, and difficulty speaking, followed by a loss of weight and jaundice. The child had normal early development. The parents of the child consulted a doctor who diagnosed the child with WD based on the clinical examination results.

The dental examination revealed no temporomandibular joint deformities, which prompted neurological consultation.

The neurological examination revealed difficulty walking with limb tremors and difficulty speaking, and the patient was referred for an MRI and laboratory results.

The laboratory results in Table 1: Clinical laboratory findings in Wilson’s disease revealed a slight elevation in transaminase and TIBC with a lower level of iron, which suggested a degree of hepatic function impairment. Serum ceruloplasmin was exceptionally low (3.1 mg/dl), and copper in 24-h urine was higher than normal. Hematology laboratory results revealed low hemoglobin, hematocrit, leukocyte, and platelet counts and a slight increase in RDW.

**Table 1:** Clinical laboratory findings in Wilson’s disease patients

*Hematology*					
Tests	Results			Reference values	Units
*Prothrombin Time*	(27/05/2024)		(14/10/2024)		
Proth. Patient	14.8		14.8		
Proth. Control	12.5		12		
Proth. Activity	83		82		
INR	1.27		1.30		
*CBC*	(27/05/2024)				
Hemoglobin	10.6		10.9	11.5–14.5	g%
Hematocrit	31.1		35.9	37–45	%
Erythrocytes	4.05		4.57	4–5.4	Mil/mm3
Leukocytes	3080		2900	4500–13,500	/mm3
Neutrophils	63.3		56.7		%
Lymphocytes	25.3		34.7		%
Monocytes	8.6		6.3		%
Eosinophils	2.6		2.3		%
*RBC’s Indices*	(27/05/2024)				
MCV	76.8		78.4	77–91	fl
MCH	26.1		23.8	25–33	pg
MCHC	34		30.3	31–37	g/100 ml
RDW	14.8		15.4	11.5–14.5	%
* Platelets Count*	55,000		45,000	150,000–400,000	/mm3
Chemistry					
Tests	Results (27/05/2024)			Reference values	Units
ALT (SGPT)	67			Up to 49	U/L
AST (SGOT)	63			Up to 49	U/L
Gamma GT	19			Up to 49	U/L
Iron	25			60–160	ug/100 ml
TIBC	546			250–410	ug/100 ml
Random urine Protein	26.8			Up to 10	mg/100 ml
Random urine Creatinine	84			20–320	
Protein/Creatinine	0.319				
Biochemistry					
Tests		Results		Reference values	Units
Ceruloplasmin		3.1		15.0–30.0	mg/dl
Copper in 24 Hour Urine		98.7		10–60	mcg/24 h

ALT alanine aminotransferase, AST aspartate aminotransferase, TIBC total iron-binding capacity, INR international normalized ratio, MCV mean corpuscular volume, MCH mean corpuscular hemoglobin, MCHC mean corpuscular hemoglobin concentration, RDW red cell distribution width

MRI revealed increased T2-weighted signal intensity in the caudate and lentiform nuclei.

Fundoscopy revealed Kayser–Fleischer (K–F) rings at the corneal limbus without any pathological features in the fundus oculi or retina, and no papilledema was observed, with a visual acuity of 9/10.

Esophagogastroduodenoscopy (EGD) revealed esophageal varices in the distal esophagus. No gastric varices were identified.

An ultrasound examination was ordered after a pediatric consultation to address the decreased weight and jaundice, which revealed a slight degree of panhepatomegaly. The hepatic parenchymal echotexture showed generalized coarseness, suggestive of parenchymal dysfunction. The gallbladder was normal in size, without calculi. Mild echogenic wall thickening was observed in the context of hepatic injury. Kidneys were normal in size and location; no calculus was observed; and no ureteric obstruction was observed. Normal thickness with normal corticomedullary differentiation was observed in renal parenchymal tissue. The spleen showed a homogeneous echotexture, with marked splenomegaly and splenic hilar enlargement. The pancreas was homogeneous in echotexture and normal in size. The urinary bladder was distended, with normal echogenicity and no wall thickening. The peritoneal cavity showed no free fluid in the abdomen or pelvis.

The study timeline is summarized in Table 2: Timeline of events.

**Table 2:** Timeline of events

Timeline of events	
2024-05-17	The patient presented to the dentist with a history of dysarthria (difficulty speaking), drooling, and looseness of the temporomandibular joint (TMJ). Dental evaluation did not identify a primary dental cause for the symptoms. The patient was referred to a neurologist for further evaluation and management of the underlying condition
2024-05-23	After physical examination the neurologist ordered: an MRI of the brain to assess for basal ganglia involvement, an EEG to evaluate for possible encephalopathy, a 24-h urine copper test to measure copper excretion, serum ceruloplasmin levels, liver function tests, and a slit-lamp examination for Kayser-Fleischer rings
2024-05-27	Biochemical examination: - Increased copper in 24-h urine excretion (98.7 mcg/24 h) and an incredibly low level of Serum Ceruloplasmin (3.1 mg/dl) - MRI: Increased T2-weighted image signals in the Caudate nucleus and Lentiform nucleus which are associated with early-stage Wilson Disease. EEG was not done due to its unavailability in the nearby hospitals
2024-05-28	After reviewing the previous results, the neurologist decided on: - An ophthalmology referral for a slit-lamp examination - An ENT referral was made to rule out middle ear pathology contributing to the patient's symptoms - A pediatrics referral to start the patient on a treatment plan
2024-06-03	- The slit lamp exam clearly showed Kayser-Fleischer rings contributing to Wilson's disease diagnosis - The ENT consultation excluded any middle ear pathologies
2024-06-06	The pediatrician referred the patient to a dietitian for copper-restricted dietary management and then prescribed the appropriate medications. A referral to physical therapy was also initiated An endoscopy was performed, revealing esophageal varices-a common complication of hepatic involvement in Wilson’s disease, and surgery was recommended
2024-07-15	In light of endoscopically confirmed esophageal varices secondary to Wilson’s disease-associated portal hypertension, the pediatrician prescribed propranolol for primary prophylaxis against variceal hemorrhage
2024-10-03	The pediatrician prescribed the patient Lactulose as prophylaxis for Hepatic Encephalopathy before undergoing surgery
2024-10-20	The patient underwent successful endoscopic variceal ligation
2025-04-04	On routine clinical evaluation, the patient demonstrated significant improvement with: - Absence of icterus (no scleral or cutaneous jaundice) - Marked improvement in motor function - Clear speech articulation with resolved dysarthria - Stable hepatic and neurological status

Timeline of patient diagnosis and management

## Discussion

WD is caused by mutations in the short arm of chromosome 13 in the ATP7B gene, resulting in copper overload in hepatocytes and copper accumulation in other tissues. More than 700 mutations are registered in the Human Gene Mutation Database. Patients are typically homozygous or compound heterozygous for pathogenic ATP7B mutations. Sequencing is a faster and less expensive method for diagnosing mutations in the ATP7B gene [4].

In this case, sequencing was not available at the hospital or nearby medical facilities; hence, it was not possible to verify the genetic mutation.

While the Leipzig score is commonly used as a diagnostic framework [5], it was not utilized in our case due to resource limitations.

Wilson disease’s hepatic manifestations usually present initially in 40% of patients [4], followed by neurologic symptoms being the second most frequent manifestation, with psychiatric manifestations affecting only approximately 10–20% of patients affected by WD [3].

In this case, the patient initially presented with psychiatric and neurologic symptoms, which started with no prior trauma or injury.

The mean age of onset of neurological symptoms is 20–30 years [4]. In this case, the patient presented with psychiatric and neurologic symptoms at the early age of 8.

Psychiatric symptoms can occur at varying times relative to the time of WD diagnosis. Symptoms include a decline in school performance, inappropriate behavior, or impulsiveness when children are diagnosed during childhood [4]. These findings were all observed in our case.

**Fig. 1:**
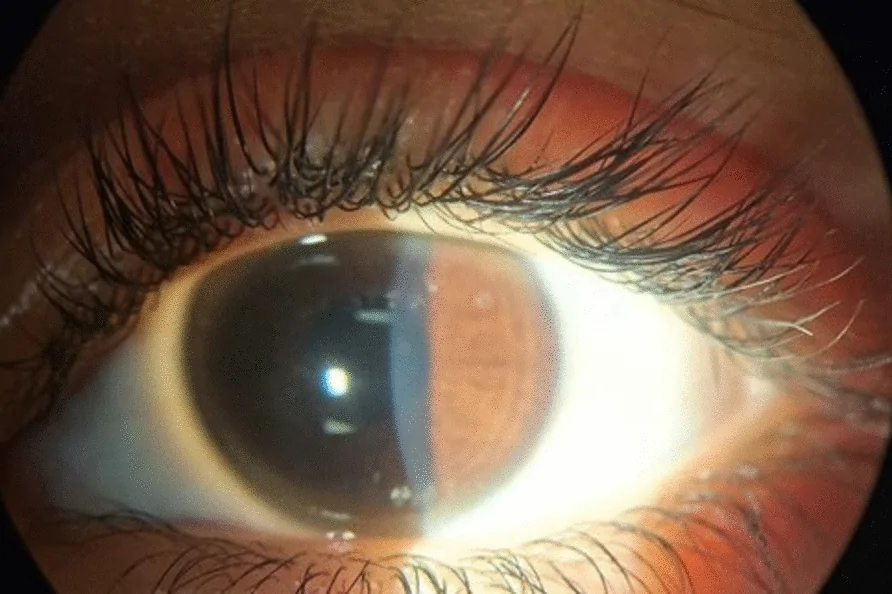
A slit lamp examination of the patient

In this case, the clinical examination revealed neurological findings associated with Wilson’s disease, prompting follow up of a slit lamp exam, which confirmed the diagnosis with the finding of Kayser–Fleischer rings. Figure 1 A slit lamp examination of the patient.

The laboratory findings in Wilson’s disease usually include increased aminotransferase levels, decreased serum ceruloplasmin levels (most common in chronic manifestations), decreased serum copper levels, increased 24-h urine copper excretion, and hypouricemia [1, 6].

In this case, the patient had an increased aminotransferase level, decreased serum ceruloplasmin levels, and elevated 24-h urine copper excretion. However, the serum copper and uric acid levels and liver functions were not measured because of financial constraints.

MRI scans in Wilson disease sometimes reveal a “double panda” sign, resulting from midbrain abnormalities, which have a “face of a giant panda” sign on T2-weighted MR images, in addition to the high-signal pontine tegmentum abnormality, which resembles a second “panda face” and hence the “double panda” signal name [7]. Brain MRI is highly suggested when neurologic Wilson disease is suspected, which usually consists of abnormalities that include symmetric hyperintense changes in T2-weighted images, which commonly affect the basal ganglia, thalami, midbrain, and pons. T1-weighted images reveal hypointense changes in advanced cases, reflecting severe tissue damage [4].

In this case, no “double panda” sign was apparent. Increased T2-weighted image signals were in the caudate nucleus and lentiform nucleus, which are associated with early stage Wilson disease.

Some studies suggest that pharmacological treatment alone is not sufficient after the onset of neurological or hepatic symptoms; the pharmacological treatments available include the copper chelators, d-penicillamine and Trientine, with the latter being more tolerated, followed by adherence monitoring [4].

WD patients with hepatic involvement usually present with jaundice, hepatomegaly, splenomegaly, ascites, esophageal or gastric varices, or hepatic encephalopathy [1]. Beta-blocker use is suggested for the prevention and treatment of esophageal varices [8]. One study indicated that lactulose administration for upper gastrointestinal bleeding reduces the incidence of hepatic encephalopathy [9].

In this case, the patient presented with jaundice, and the ultrasound findings included hepatomegaly and splenomegaly. Esophageal varices were observed via endoscopy, and propranolol was first prescribed for their prevention and treatment. The patient was then given lactulose as a preventive measure for hepatic encephalopathy before undergoing successful endoscopic variceal ligation surgery.

The dietary copper restriction could help manage excess copper as an adjunctive therapy, which can reduce further liver injury that might lead to liver cirrhosis and the need for a liver transplant. To our knowledge, no clinical studies have been conducted on neurological WD treatment [4].

In this case, the patient was initially treated with the copper chelator penicillamine, which was the only available option in the treatment center, and a dietary copper restriction, followed by the implementation of a physical therapy plan.

A delayed diagnosis following the onset of symptoms is usually an indicator of a poor prognosis [4].

In this case, using the appropriate treatment options had incredibly positive results, with the patient showing improvements at every checkup and a healthy recovery.

Owing to financial constraints, the patient was monitored clinically by a pediatrician without further specialized investigations.

## Conclusions

This case highlights the diagnostic challenges of pediatric Wilson’s disease (WD), particularly when early psychiatric and neurologic symptoms are present in an 8-year-old male. The patient’s hepatic dysfunction (elevated transaminases, low ceruloplasmin, hepatomegaly) and neurologic signs (dysarthria, MRI changes in the basal ganglia) underscore the multisystem nature of WD. The presence of Kayser-Fleischer rings and urinary copper excretion confirmed the diagnosis, despite limited genetic testing resources. Early intervention with penicillamine and dietary copper restriction led to significant clinical improvement, emphasizing the importance of timely diagnosis in preventing irreversible liver or neurologic damage. This case adds to the scarce literature on WD in pediatric populations, particularly in resource-limited settings, and reinforces the need for heightened awareness of atypical presentations.

## Data Availability

All clinical data supporting the conclusions of this article are included within the manuscript and its tables and figures. No additional datasets were generated or analyzed.
